# Evaluating the Impact of an Online Mindfulness Program on Healthcare Workers in Korean Medicine Institutions: A Two-Year Retrospective Study

**DOI:** 10.3390/healthcare12222238

**Published:** 2024-11-10

**Authors:** Chan-Young Kwon

**Affiliations:** Department of Oriental Neuropsychiatry, Dong-Eui University College of Korean Medicine, Busan 47227, Republic of Korea; beanalogue@naver.com; Tel.: +82-51-850-8808

**Keywords:** healthcare workers, Korean medicine, mindfulness, hwa-byung

## Abstract

**Background/Objectives**: This retrospective study evaluated the effectiveness of a two-year online mindfulness program (five biweekly sessions) combined with a smartphone application for healthcare workers (HCWs) in Korean medicine (KM) institutions. **Methods**: Twenty-three participants, including KM University students, KM doctors, and nurses, completed a 9-week online mindfulness program in 2023 or 2024. The pre- and post-intervention surveys assessed subjective health status (SHS), knowledge of mind–body modalities (MBMs), hwa-byung (HB) symptoms, emotional labor (EL), burnout, and program satisfaction. **Results**: Participants showed significant improvements in SHS (*p* = 0.008) and MBM knowledge (*p* = 0.035). HB personality scores decreased significantly (*p* = 0.027), while the reduction in HB symptoms approached statistical significance (*p* = 0.052). The frequency of interactions among job-focused EL increased (*p* = 0.003). The subgroup analysis revealed significant reductions in HB personality traits (*p* = 0.017) and symptoms (*p* = 0.006) among practicing KM doctors and nurses. No significant changes were observed in burnout levels. Participants reported high satisfaction (median 8.00 [IQR 8.0–9.0]) and willingness to recommend the program (median 5.00 [IQR 4.0–5.0]). **Conclusions**: Analysis of the 2-year results suggests that the online mindfulness program effectively improved SHS, MBM knowledge, and HB-related symptoms among HCWs in KM institutions, particularly among practicing professionals. High satisfaction rates indicated the acceptability of the program. Future research should use larger sample sizes and randomized controlled designs to further validate these findings and explore long-term outcomes. This intervention shows promise as a tool to promote mental health in Korean healthcare settings.

## 1. Introduction

Healthcare workers (HCWs) are exposed to mental health threats’ and burnout due to their occupations is associated with high levels of job stress. According to a recent survey of approximately 1000 doctors and nurses in South Korea, more than 80% of the respondents were exposed to work-related burnout and emotional difficulties due to work [1]. In the Korean medical environment, which is divided into conventional Western medicine (WM) and Korean medicine (KM), studies on the mental health status of HCWs working in KM institutions are important from a public health perspective. A previous review study analyzed 11 studies investigating the mental health of nurses working in KM hospitals and identified differences between individuals in WM and KM hospitals, some of which included relatively lower job satisfaction among KM nurses [2].

The work environment of HCWs, combined with Korea’s unique cultural environment, may increase their vulnerability to hwa-byung (HB), a type of anger-related culture-bound syndrome that occurs primarily in relation to emotional suppression [3]. HB is defined as a syndrome in which unresolved negative emotions, including anger, erupt in the form of “fire-like” symptoms [4]. The prevalence of HB in the general population is known to be 4–13.3% [4], but it may be higher in HCWs exposed to job stress. For example, a recent survey found that approximately 20% of nurses working in KM institutions were exposed to HB [5]. Mental health problems in this population are important because they not only affect individual well-being and quality of life but also potentially affect patient outcomes [6]. Importantly, medical errors that occur in KM clinics, which include acupuncture needle-related adverse events (e.g., missing needle removal), can potentially pose serious threats to patient health [7]. A study of nurses working in KM clinics found that the presence of HB was associated with higher levels of depression and cognitive failure [5], potentially associated with medical errors [8]. Therefore, strategies to improve mental health in this population are urgently needed. This need has been highlighted in the context of the coronavirus disease 2019 pandemic [9].

Mind–body modalities (MBMs) represent a group of interventions that focus on the interactions among the brain, mind, body, and behavior, with the intent to use the mind to affect physical functioning and promote health [10]. These modalities encompass various practices including meditation, yoga, tai chi, qigong, relaxation techniques, and breathing exercises. Among these, mindfulness meditation has emerged as one of the most researched approaches in healthcare settings [11,12]. Mindfulness meditation is characterized as the practice of maintaining attention on the present-moment experience with an attitude of openness, curiosity, and non-judgment [13]. This practice typically involves two core components: (1) the self-regulation of attention to maintain the focus on the immediate experience, and (2) approaching one’s experiences with openness, curiosity, and acceptance [13].

While online mindfulness programs gained prominence during the coronavirus disease 2019 pandemic, their relevance extends well beyond the pandemic era for several compelling reasons. First, HCWs in KM institutions often face challenging work schedules that make in-person attendance difficult [5]. The online format provides flexible access to mental health support without compromising their clinical responsibilities or requiring additional commuting time [14]. Second, the combination of synchronized online sessions and a smartphone application enables continuous engagement and practice between sessions, which is crucial for developing sustainable mindfulness habits. Finally, the cost-effectiveness and scalability of online programs make them particularly attractive for healthcare institutions seeking to implement staff wellness programs. These advantages of online delivery remain relevant regardless of the pandemic having eased, particularly in the Korean healthcare context where work-related stress and burnout continue to be significant concerns [1].

## 2. Methods

### 2.1. Objective

The current study aimed to evaluate the effectiveness of this program for improving the mental health of HCWs working in KM institutions through statistical analysis of outcome measures collected from participants over the past two years (2023–2024).

### 2.2. Study Design

This study was a retrospective analysis of the two-year results of a mindfulness program that was previously implemented annually. At this stage, the focus was not only on gathering initial evidence of effectiveness but also on evaluating the program’s feasibility and identifying potential areas for content modification. Although data were collected over two years, due to the small sample size and similar program implementation across both years, I analyzed the combined data to increase the statistical power. A year-by-year analysis was not performed due to the limited sample size for each year. Although this program had been implemented annually since 2022, only the results of 2023 and 2024 were analyzed due to the homogeneity of the evaluations conducted in the program. Additionally, since the meditation smartphone application developed had been available since 2023, it was judged that there was a difference in the intensity of the program provided in 2022 and that of the program provided from 2023.

### 2.3. Participants

**Figure 1:**
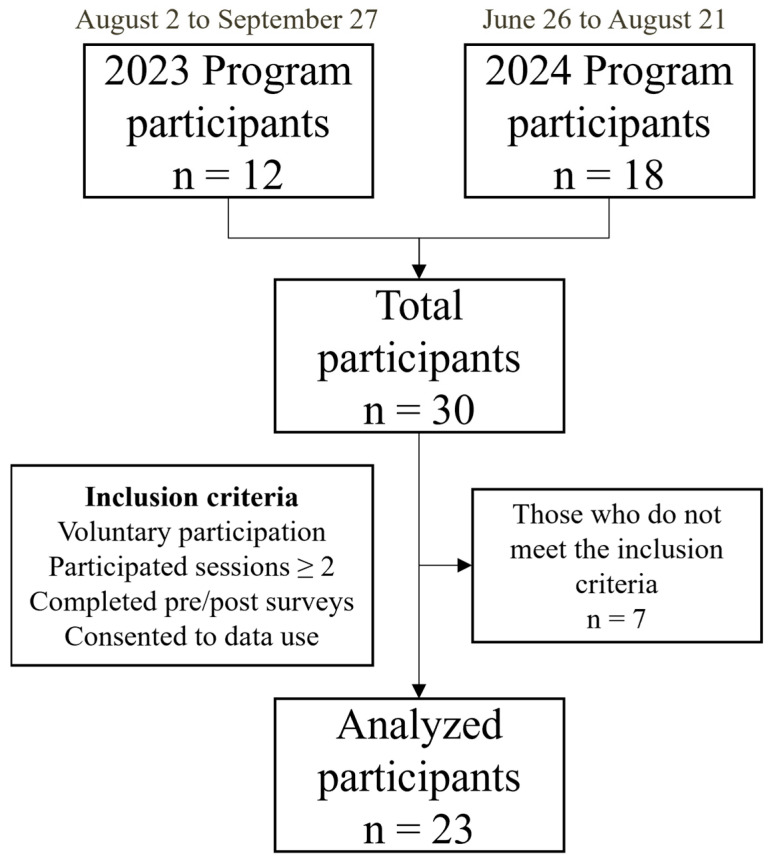
Flow diagram of this study.

This program included HCWs of KM institutions. Although primarily designed for KM HCWs, the participation was also open to third- and fourth-year students from KM universities. Participants were recruited through social media announcements distributed through the branch directors of the Busan Association of Korean Medicine, along with promotional posts on the website of a Korean medicine college affiliated hospital. In 2023 and 2024, 12 and 18 individuals participated in this program, respectively. Of these, 23 participants who met the following inclusion criteria were included in the final analysis (Figure 1): (1) participation in the mindfulness program in 2023 (2 August to 27 September) or 2024 (26 June to 21 August); (2) voluntary participation after being informed that the program participation results would be used for research purposes; (3) participation in more than two sessions of the program; (4) completion of two anonymous online surveys before and after the program implementation; and (5) consent to the collection and use of personal information (e.g., age group, sex) for data analysis purposes.

### 2.4. Delivery of the Mindfulness Program

The research team developed an online mindfulness program and a smartphone application to improve the mental health of HCWs in KM clinics and reported the results of its application as a case series annually [14]. This program differs from the existing representative mindfulness program, the mindfulness-based stress reduction (MBSR) program, in that it sets the ratio of mindfulness training to loving–kindness at 1:1. This setting encourages deep-acting EL related to reducing job stress, considering the characteristics of the HCWs’ jobs [14].

The mindfulness program was delivered online for 9 weeks. The program consisted of five biweekly sessions, each lasting 2 h, conducted from 8 PM to 10 PM to accommodate the participants’ work schedules. Real-time online delivery was facilitated using Naver’s Whale-on platform. Naver’s Whale-on is a South Korean online video conferencing platform that provides real-time interactive features including screen sharing, chat functions, and breakout rooms. This platform was chosen for its stability and familiarity to Korean users.

**Figure 2:**
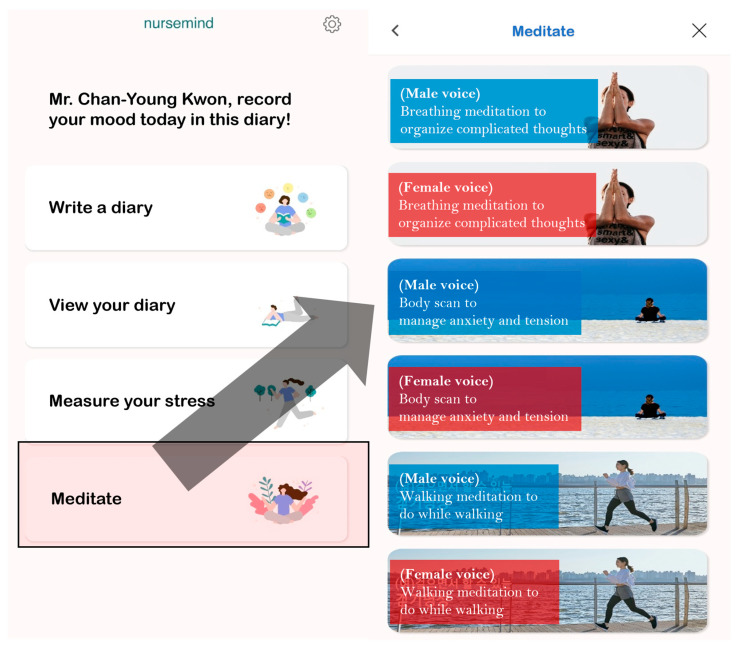
Smartphone application that participants were encouraged to use. **Note**. The participants can choose any type of content and practice mindfulness for 10–20 min whenever they want. This application provides the following types of content in male and female versions: breathing meditation, body scan, walking meditation, eating meditation, loving–kindness meditation, forgiveness meditation, progressive muscle relaxation, autogenic training, and five meditative music tracks.

The structure and content of this program were detailed in the previous case series [14]. Similarly to MBSR programs, the intervention incorporated homework assignments to encourage daily mindfulness practice. To support this, participants were provided with a specially developed smartphone application that contained mindfulness meditation content, which served as a tool for daily assignments and training exercises between formal online sessions. Specifically, participants were asked to complete daily mindfulness meditation practice using the provided smartphone application (10–20 min per day) (Figure 2).

### 2.5. Data Collection

Participants were free to participate in the survey through a free online survey platform (Moaform, http://www.moaform.com, accessed on 1 October 2024), before and after the program. Specifically, the pre-survey was completed within a week before participating in the program, and the post-survey was completed within a week after participating in the program. To minimize social desirability bias in survey responses [15], several methodological safeguards were implemented. All surveys were conducted anonymously through the online platform. Participants received written assurance that their responses would remain confidential and would not affect their relationship with the program or institution. Survey administration was managed by a research assistant who was not involved in program delivery. Furthermore, participants were explicitly encouraged to provide honest feedback, emphasizing that critical responses would be valuable for program improvement.

### 2.6. Outcomes

The outcome measures included were as follows: sociodemographic characteristics (gender, sex, occupation, clinical experience), subjective health status (SHS) (1 point: very bad, 2 points: bad, 3 points: average, 4 points: good, 5 points: very good), knowledge and attitude toward MBMs (5-point Likert scale), previous participation in other mindfulness programs, HB scale including HB personality (HB-P) and HB symptoms (HB-S) [16], the emotional labor (EL) scale by Lee, including employee-focused EL and job-focused EL [17], the Copenhagen Burnout Inventory (CBI) (Korean version) [18], experience of medical errors within the past 2 months, and satisfaction and dissatisfaction with participation in this program. The HB scale evaluates the vulnerability to HB with 16 items on the HB-P and the severity of psychological and somatic symptoms of HB with 15 items on the HB-S [16]. A score of 30 or higher on the HB-S suggests the presence of HB [16]. The EL scale by Lee was built on Grandey’s conceptual model of EL [19] and consists of six items to assess employee-focused EL and eight items to assess job-focused EL [17]. The Korean version of the CBI is based on the CBI of Kristensen et al. [20], and this tool consists of three domains: personal burnout (seven items), work-related burnout (six items), and client-related burnout (six items), which assess burnout from various perspectives [18]. Program satisfaction was evaluated using a structured survey that included overall satisfaction (10-point Likert scale) and future engagement intentions (5-point Likert scales for program participation and recommendation intentions). This survey was developed specifically for this study and administered post-intervention through the same online platform used for other outcome measures.

### 2.7. Data Analysis

Statistical analyses were performed using SPSS software (version 18.0; SPSS Inc., Chicago, IL, USA). Descriptive statistics were used to analyze demographic characteristics (age group, sex, occupation, and clinical experience), previous participation in other mindfulness programs, and experience with medical errors. To assess the normality of the distribution of continuous variables (SHS, knowledge and attitudes toward MBMs, HB personality and symptoms, EL, and burnout), the Shapiro–Wilk test was conducted, as it provides better power to detect non-normality in small samples. For variables that met the assumption of normality, paired *t*-tests were used to compare the results pre- and post-intervention. When the normality assumption was violated, Wilcoxon signed-rank tests were used as non-parametric alternatives. Subgroup analyses were performed to distinguish between KM University students and medical staff, including KM doctors (KMDs) and nurses. Differences were considered statistically significant for all analyses. Given the small sample size (n = 23), I not only examined statistical significance but also calculated the number and percentage of participants showing improvements in each outcome measure to provide a more comprehensive understanding of the program’s effects. This approach allows for a more nuanced interpretation of the results, particularly for outcomes showing statistical significance despite the limited sample size.

For the Likert scale variables, I report median (interquartile range [IQR]) values considering the ordinal nature of these measurements. While some researchers argue for treating Likert scales as ordinal data, others suggest that parametric statistics can be used under certain conditions [21,22]. I used non-parametric Wilcoxon signed-rank tests for these variables as they should be treated as ordinal rather than continuous data, and I interpreted the results with appropriate caution regarding the limitations of Likert scale measurements.

### 2.8. Ethical Considerations

All participants provided written informed consent before study enrollment. The consent form included information about the study purpose, procedures, potential risks and benefits, data usage, and participants’ rights. Participants were informed that their data would be used for research purposes and that they could withdraw at any time without penalty. The consent process was conducted online through a secure platform.

## 3. Results

### 3.1. Characteristics of the Participants

The sample showed considerable demographic diversity. Of the 23 participants, 18 (78.3%) were female. The age distribution was notably wide, with the majority being under 30 years (17 individuals, 73.9%), followed by those aged ≥50 years (three individuals, 13.0%), those in their thirties (two individuals, 8.7%), and those in their forties (one individual, 4.3%). The participants represented different roles within KM settings: thirteen were KM University students (56.5%), six were KMDs (26.1%), three were nurses (13.0%), and one was a KM hospital administrator (4.3%). Among practicing professionals (KMDs and nurses), clinical experience varied substantially, ranging from 5 to 444 months (mean = 160.00 ± 169.19 months) (Table 1). Given this heterogeneity, I conducted the analyses in two ways: first, examining the overall effects across all participants, and second, performing subgroup analyses comparing students and comparing practicing professionals to account for their different backgrounds and experiences (Table 2, Table 3 and Table 4). This analytical approach allows us to better understand how the program’s effects might vary across different participant subgroups.

### 3.2. Changes in All Participants

Among all participants (n = 23), there was a statistically significant improvement in SHS, with mean scores increasing from 3.26 ± 0.75 before the program to 3.70 ± 0.63 after the program (t = 2.67, *p* = 0.008). This improvement was observed in 10 participants (43.5%), while 12 (52.2%) showed no change and 1 (4.3%) showed a slight decrease. Furthermore, in terms of MBM, the participants showed a significant increase in knowledge, from a median 3.00 (IQR 2.0–3.0) pre-intervention to a median 3.00 (IQR 3.0–4.0) post-intervention (Z = 2.11, *p* = 0.035). Individual changes showed that 11 participants (47.8%) reported increased knowledge, 9 (39.1%) remained unchanged, and 3 (13.0%) reported decreased knowledge. A notable reduction in HB-P scores was observed in 22 participants (95.7%), from 29.17 ± 8.61 to 26.22 ± 11.43 (Z = −2.37, *p* = 0.027). Similarly, 22 participants (95.7%) showed decreased HB-S scores from 18.13 ± 9.03 to 15.39 ± 11.25, although this change was only marginally significant (Z = −2.06, *p* = 0.052). In terms of job-focused EL, a significant increase was found in the frequency of interactions among job-focused EL, with scores rising from 3.28 ± 0.92 pre-program to 3.77 ± 0.50 post-program (Z = 3.02, *p* = 0.003), observed in 12 participants (52.2%) (Table 2).

### 3.3. Changes Among KM University Students

Among KM University students (n = 13), SHS increased from 3.38 ± 0.77 to 3.77 ± 0.73, with improvements observed in five participants (38.5%), although this change was not statistically significant (Z = 1.67, *p* = 0.096). There was a significant improvement in MBM knowledge, with scores increasing from median 3.00 (IQR 3.0–3.0) to median 3.00 (IQR 3.0–4.0) (Z = 2.24, *p* = 0.025), with five students (38.5%) showing improvement. While all students (100%) showed decreased HB-P scores and most (12, 92.3%) showed decreased HB-S scores, these reductions were not statistically significant (Table 3).

### 3.4. Changes Among KMDs and Nurses

In the group of KMDs and nurses (n = 9), there was a significant reduction in HB-P scores, from 28.33 ± 10.15 to 23.00 ± 8.12 (Z = −3.51, *p* = 0.017), with improvements observed in eight participants (88.9%). Additionally, HB-S showed a significant decrease from 18.00 ± 11.15 to 10.5 ± 8.02 (Z = −4.61, *p* = 0.006), with all participants (100%) showing an improvement (Table 4). Four of them (44.4%) reported experiencing medical errors in the 2 months prior to participating in the program, and three (33.3%) reported experiencing medical errors in the 2 months following participation in the program.

### 3.5. Participants’ Satisfaction with the Program

The participants generally expressed high satisfaction with the mindfulness program and smartphone application. On a 10-point Likert, the median satisfaction score was 8.00 (IQR 8.0–9.0), indicating that most of the participants found the program helpful. Specifically, KM University students rated the program at 8.00 (IQR 8.0–9.0), and KMDs rated it at 8.00 (IQR 7.75–8.5). When asked if they would participate in a similar program in the future, their median response was 4.00 (IQR 4.0–5.0) on a 5-point Likert scale. Furthermore, participants indicated a willingness to continue using the smartphone application provided (median 4.00 [IQR 4.0–5.0]) and to recommend both the program and the application to their colleagues, with scores of 5.00 (IQR 4.0–5.0) and 4.00 (IQR 4.0–5.0), respectively (Table 5).

## 4. Discussion

### 4.1. Findings of This Study

This study evaluated the effectiveness of an online mindfulness program combined with a smartphone application for HCWs in KM institutions. The results show several significant improvements in mental health and well-being of the participants and highlight areas for further investigation and program refinement.

### 4.2. Clinical Interpretation of the Findings

The significant improvement in SHS among all participants (observed in 43.5% of participants) suggests that the program positively affected their overall well-being. This finding is consistent with those of a previous study on the benefits of mindfulness interventions for healthcare professionals [23]. The increased knowledge of MBMs, reported by 47.8% of participants, indicates that the program successfully educated the participants, which could enable them to incorporate these MBM techniques into their daily lives and professional practice. This change in knowledge about MBM suggests long-term benefits [24]. One of the most notable findings was the significant reduction in HB-P scores (observed in 95.7% of participants) and a marginally significant decrease in HB-S (also in 95.7% of participants). This remarkably high proportion of participants showing improvement is particularly relevant given the prevalence of HB among nurses in KM institutions [5]. The program’s capacity to address this culture-bound syndrome suggests its potential as a targeted intervention for specific mental health issues in the Korean healthcare context, especially from the perspective of anger suppression [25,26].

The significant increase in the frequency of interactions among job-focused EL is an intriguing finding that warrants further investigation. These results contrast with those of a recent meta-analysis that found short-term positive effects of mindfulness-based interventions on burnout among nurses [27]. However, in this study, I observed that one subscale of job-focused EL (frequency of interactions) increased in the participants. This could be due to environmental changes in participants over time or changes in their perceptions of their work environment. Specifically, it is possible that increased mindfulness made participants more aware of their emotional regulation efforts at work [28]. Although mindfulness training itself appears to rarely cause serious adverse effects, increased mindfulness may help participants confront the psychological distress they experience [29]. Alternatively, this could indicate the need to refine the program to better address the challenges of EL in healthcare settings. This is also a disappointing result, considering the results of previous studies [30], in which no significant changes in participants’ burnout levels were observed. Although improving burnout in HCWs through MBM may require long-term intervention [31], these results highlight the need to revise the program.

The subgroup analysis revealed interesting differences among KM University students, KMDs, and nurses. Significant improvements in HB-P and HB-S scores among practicing professionals but not among students suggest that the program may be particularly beneficial for those actively involved in patient care. This could be due to the higher levels of stress and emotional demands experienced by practicing HCWs [32], which make them more receptive to the benefits of mindfulness practice [33]. High satisfaction scores and the willingness to recommend the program and its application to colleagues are encouraging indicators of the acceptability and perceived value of the intervention. This positive reception suggests that the program has the potential for a wider implementation and long-term engagement. The findings have several practical implications for healthcare institutions and policymakers. The effectiveness of the program in reducing HB symptoms suggests that culturally tailored mindfulness interventions could be valuable additions to employee wellness programs in Korean healthcare settings. The high satisfaction rates and ease of online delivery make this intervention feasible for widespread implementation. Moreover, the potential of the program to improve SHS and increase the knowledge of MBMs could contribute to more resilient and well-informed HCWs. This may improve patient care in KM institutions [7].

### 4.3. Limitations

This study has several limitations. First, the small sample size (n = 23) means careful interpretation is required of the statistical significance findings. While I observed statistically significant improvements in several outcomes, the small sample size means that changes in a few participants could have substantially influenced the statistical results. To provide more context, I have reported the number of participants showing improvements for each significant outcome. However, larger studies are needed to confirm these findings and establish their generalizability. While the lack of a control group is a limitation, it reflects the developmental stage of this research. The program currently requires both repeated validation of its effectiveness and evaluation of its feasibility, with the possibility of content modifications based on participant responses and outcomes. I am systematically accumulating evidence, including the current findings, to refine the program content and structure. This careful developmental approach will inform the design of a future prospective controlled trial with a larger sample size. Second, the self-reported nature of the results and the potential for response bias should be considered. Incorporating objective measures of stress (e.g., heart rate variability) and performance metrics could provide more robust evidence of the impact of the program. Also, despite implementing several methodological safeguards to minimize social desirability bias in the surveys (including anonymity, third-party administration, and explicit encouragement of honest feedback), I acknowledge that such bias cannot be completely eliminated in self-reported outcomes [15]. This is particularly relevant given the positive relationship between participants and the program providers in the KM community. Future studies should consider incorporating more objective outcome measures, such as physiological indicators or independent behavioral observations, to complement self-reported data. Additionally, implementing a mixed-methods approach with qualitative interviews by independent researchers might provide more nuanced insights into participants’ experiences while helping to validate self-reported outcomes. Third, the benefits identified by the participants in this study were assessed in the short term. Longitudinal follow-up studies are valuable for assessing the long-term effects of interventions and determining whether the benefits are sustained over time. Fourth, this study was conducted quantitatively and did not explore the qualitative experiences of individual participants when they accessed the MBM program. Further qualitative research could provide deeper insight into the experiences of the participants and help identify areas for program improvement. Fourth, a methodological limitation of this study concerns the use of Likert scales for outcome measurements. While Likert scales are widely used in behavioral and social science research, there is ongoing debate about their statistical treatment and interpretation [21,22]. While I addressed the ordinal nature of these data by using appropriate non-parametric statistical methods and reporting both means and medians, future studies might benefit from using continuous measurement scales or alternative assessment tools to obtain more robust quantitative data. Finally, the heterogeneous nature of the sample creates challenges for interpretation. The varying levels of clinical experience, different professional roles, and wide age range may have influenced how participants engaged with and benefitted from the program. However, this diversity also provides valuable insights into how the program might be adapted for different subgroups within healthcare settings.

## 5. Conclusions

This analysis of two-year results has shown significant improvements in SHS, MBM knowledge, and HB symptoms among HCWs in KM institutions. The quantitative findings support the effectiveness of this online mindfulness program. While further research is needed to address this study’s limitations and explore long-term outcomes, the results suggest that this intervention could offer a valuable tool in promoting the mental health of healthcare workers in Korea and potentially in other cultural contexts.

## Figures and Tables

**Table 1:** The basic information of the participants.

Variables	Options	KM University Students (n = 13)	KMDs (n = 6)	Nurses (n = 3)	Other (n = 1)
Age	<30	13	4	0	0
	30–39	0	1	0	1
	40–49	0	1	0	0
	>50	0	0	3	0
Sex	Male	2	3	0	0
	Female	11	3	3	1
Clinical experience	Mean (range)	NA	6 mon (5 to 264 mon)	3 mon (153 to 444 mon)	NA
Experience participating in a meditation program	Yes	2	1	0	0
	No	11	5	3	1
N of sessions attended	2	1	0	0	0
	3	3	0	0	0
	4	5	0	0	0
	5	4	6	3	1

**Abbreviations**: KM, Korean medicine; KMD, Korean medicine doctor; NA, not applicable.

**Table 2:** Changes after program participation among all participants.

Variables	Before, Mean (SD) or Median [IQR]	After, Mean (SD) or Median [IQR]	Improved, n (%)	MD (SD)	t or Z-Value ^§^	*p*-Value
SHS	3.26 (0.75)	3.70 (0.63)	10 (43.5%)	0.43 (0.66)	2.67 ^§^	0.008 **
*“How much do you think you know about MBM?”* (5-point Likert)	3 [3]	3 [3, 4]	7 (30.4%)	NA	2.11 ^§^	0.035 *
*“Do you think MBMs could help improve your mental health?”* (5-point Likert)	5 [4, 5]	5 [4, 5]	3 (13.0%)	NA	0.45 ^§^	0.655
*“Do you think a smartphone application that offers MBMs could help improve your mental health?”* (5-point Likert)	4 [4]	4 [4, 5]	8 (34.8%)	NA	1.94 ^§^	0.052
*“Would you be willing to use a smartphone application that offers MBMs to improve your mental health?”* (5-point Likert)	4 [4, 5]	4 [4, 5]	5 (21.7%)	NA	0.33 ^§^	0.739
1. HB-P	29.17 (8.61)	26.22 (11.43)	22 (95.7%)	−2.96 (5.97)	−2.37	0.027 *
2. HB-S	18.13 (9.03)	15.39 (11.25)	22 (95.7%)	−2.74 (6.39)	−2.06	0.052
3. EL	3.09 (0.81)	3.17 (0.49)	15 (65.2%)	0.09 (0.67)	0.62	0.543
3.1. Employee-focused EL	2.98 (0.93)	2.89 (0.86)	7 (30.4%)	−0.09 (0.81)	−0.52	0.608
3.1.1. Surface acting	2.88 (0.92)	2.68 (0.88)	5 (21.7%)	−0.20 (0.92)	−1.05	0.304
3.1.2. Deep acting	3.07 (0.98)	3.10 (0.97)	7 (30.4%)	0.03 (0.88)	0.20 ^§^	0.840
3.2. Job-focused EL	3.17 (0.81)	3.39 (0.38)	7 (30.4%)	0.28 (0.73)	1.25 ^§^	0.210
3.2.1. Frequency of interactions	3.28 (0.92)	3.77 (0.50)	12 (52.2%)	0.49 (0.71)	3.02 ^§^	0.003 **
3.2.2. Duration of interactions	2.96 (0.99)	2.93 (0.73)	2 (8.7%)	−0.02 (1.27)	0.08 ^§^	0.938
3.2.3. Variety of expressions	3.20 (0.95)	3.30 (0.56)	2 (8.7%)	0.10 (0.77)	0.06 ^§^	0.954
4. Burnout	2.46 (0.48)	2.40 (0.57)	5 (21.7%)	−0.06 (0.36)	−0.85	0.402
4.1. Personal	2.76 (0.49)	2.63 (0.47)	17 (73.9%)	−0.12 (0.34)	−1.74	0.096
4.2. Work	2.43 (0.62)	2.29 (0.66)	5 (21.7%)	−0.14 (0.61)	−1.14	0.267
4.3. Client	2.15 (0.71)	2.14 (0.76)	20 (87.0%)	−0.01 (0.50)	−0.07	0.945

**Abbreviations**: EL, emotional labor; HB-P, hwa-byung personality; HB-S, hwa-byung symptoms; HB, hwa-byung; IQR, interquartile range; MBM, mind–body modality; MD, mean difference; NA, not applicable; SD, standard deviation; SHS, subjective health status. **Note**: ^§^, Wilcoxon signed-rank test is performed. *, *p* < 0.05, **, *p* < 0.01.

**Table 3:** Changes after program participation among KM University students.

Variables	Before, Mean (SD) or Median [IQR]	After, Mean (SD) or Median [IQR]	Improved, n (%)	MD (SD)	t or Z-Value ^§^	*p*-Value
SHS	3.38 (0.77)	3.77 (0.73)	5 (38.5%)	0.38 (0.77)	1.67 ^§^	0.096
*“How much do you think you know about MBM?”* (5 point-Likert)	3 [3, 3]	3 [3, 4]	5 (38.5%)	NA	2.24 ^§^	0.025 *
*“Do you think MBMs could help improve your mental health?”* (5-point Likert)	5 [4, 5]	5 [4, 5]	2 (15.4%)	NA	1.41 ^§^	0.157
*“Do you think a smartphone application that offers MBMs could help improve your mental health?”* (5-point Likert)	4 [4]	4 [4, 5]	4 (30.8%)	NA	1.00 ^§^	0.317
*“Would you be willing to use a smartphone application that offers MBMs to improve your mental health?”* (5-point Likert)	4 [4]	4 [4, 5]	2 (15.4%)	NA	0.00 ^§^	1.000
1. HB-P	27.54 (7.04)	25.23 (11.76)	13 (100%)	−2.31 (6.94)	−1.20	0.254
2. HB-S	16.62 (8.57)	14.62 (10.98)	12 (92.3%)	−2.00 (6.38)	−1.13	0.280
3. EL	2.98 (0.66)	2.97 (0.49)	9 (69.2%)	−0.01 (0.56)	0.08 ^§^	0.937
3.1. Employee-focused EL	2.88 (0.82)	2.65 (0.91)	2 (15.4%)	−0.23 (0.73)	−1.14	0.277
3.1.1. Surface acting	2.72 (0.83)	2.51 (0.90)	1 (7.7%)	−0.21 (0.93)	−0.80	0.441
3.1.2. Deep acting	3.05 (0.89)	2.79 (1.04)	4 (30.8%)	−0.26 (0.68)	1.45 ^§^	0.147
3.2. Job-focused EL	3.05 (0.71)	3.21 (0.35)	4 (30.8%)	0.16 (0.67)	0.63 ^§^	0.529
3.2.1. Frequency of interactions	3.23 (0.86)	3.62 (0.54)	5 (38.5%)	0.38 (0.54)	2.26 ^§^	0.024 *
3.2.2. Duration of interactions	2.73 (0.93)	2.77 (0.60)	2 (15.4%)	0.04 (1.20)	0.12	0.910
3.2.3. Variety of expressions	3.08 (0.80)	3.10 (0.48)	1 (7.7%)	0.03 (0.73)	0.31 ^§^	0.757
4. Burnout	2.50 (0.44)	2.38 (0.56)	2 (15.4%)	−0.12 (0.36)	−1.20	0.252
4.1. Personal	2.82 (0.44)	2.64 (0.47)	9 (69.2%)	−0.19 (0.38)	−1.77	0.101
4.2. Work	2.53 (0.58)	2.36 (0.66)	2 (15.4%)	−0.17 (0.67)	−0.89	0.390
4.3. Client	2.09 (0.73)	2.08 (0.82)	10 (76.9%)	−0.01 (0.52)	−0.09	0.931

**Abbreviations**: EL, emotional labor; HB-P, hwa-byung personality; HB-S, hwa-byung symptoms; HB, hwa-byung; IQR, interquartile range; MBM, mind–body modality; MD, mean difference; NA, not applicable; SD, standard deviation; SHS, subjective health status. **Note**: ^§^, Wilcoxon signed-rank test is performed. *, *p* < 0.05.

**Table 4:** Changes after program participation among KMDs and nurses.

Variables	Before, Mean (SD) or Median [IQR]	After, Mean (SD) or Median [IQR]	Improved, n (%)	MD (SD)	t or Z-Value ^§^	*p*-Value
SHS	3.00 (0.89)	3.50 (0.55)	4 (44.4%)	0.50 (0.55)	2.24	0.076
*“How much do you think you know about MBM?”* (5-point Likert)	3 [2, 4]	4 [2, 4]	2 (22.2%)	NA	1.34 ^§^	0.180
*“Do you think MBMs could help improve your mental health?”* (5-point Likert)	5 [4, 5]	5 [4, 5]	1 (11.1%)	NA	0.00 ^§^	1.000
*“Do you think a smartphone application that offers MBMs could help improve your mental health?”* (5-point Likert)	4 [4, 5]	5 [4, 5]	4 (44.4%)	NA	1.41 ^§^	0.157
*“Would you be willing to use a smartphone application that offers MBMs to improve your mental health?”* (5-point Likert)	4 [4, 5]	5 [4, 5]	3 (33.3%)	NA	0.58 ^§^	0.564
1. HB-P	28.33 (10.15)	23.00 (8.12)	8 (88.9%)	−5.33 (3.72)	−3.51	0.017 *
2. HB-S	18.00 (11.15)	10.5 (8.02)	9 (100%)	−7.50 (3.99)	−4.61	0.006 **
3. EL	3.13 (1.27)	3.45 (0.36)	6 (66.7%)	0.32 (1.03)	0.76	0.481
3.1. Employee-focused EL	3.03 (1.35)	3.25 (0.66)	4 (44.4%)	0.22 (1.04)	0.31 ^§^	0.753
3.1.1. Surface acting	3.05 (1.32)	2.83 (0.84)	3 (33.3%)	−0.22 (1.09)	−0.49	0.643
3.1.2. Deep acting	3.00 (1.41)	3.67 (0.67)	2 (22.2%)	0.67 (1.17)	0.95 ^§^	0.344
3.2. Job-focused EL	3.21 (1.21)	3.61 (0.35)	2 (22.2%)	0.40 (1.11)	0.87	0.423
3.2.1. Frequency of interactions	3.22 (1.29)	4.00 (0.37)	6 (66.7%)	0.78 (1.07)	1.79	0.134
3.2.2. Duration of interactions	3.33 (1.21)	3.17 (0.82)	0 (0%)	−0.17 (1.86)	−0.22	0.835
3.2.3. Variety of expressions	3.11 (1.44)	3.50 (0.69)	0 (0%)	0.39 (1.10)	0.86	0.427
4. Burnout	2.26 (0.60)	2.14 (0.44)	3 (33.3%)	−0.12 (0.33)	0.94 ^§^	0.345
4.1. Personal	2.45 (0.45)	2.40 (0.37)	7 (77.8%)	−0.05 (0.31)	−0.38	0.721
4.2. Work	2.25 (0.85)	1.92 (0.51)	3 (33.3%)	−0.33 (0.57)	−1.44	0.210
4.3. Client	2.06 (0.85)	1.97 (0.71)	9 (100%)	−0.08 (0.42)	−0.49	0.646

**Abbreviations**: EL, emotional labor; HB-P, hwa-byung personality; HB-S, hwa-byung symptoms; HB, hwa-byung; IQR, interquartile range; MBM, mind–body modality; MD, mean difference; NA, not applicable; SD, standard deviation; SHS, subjective health status. **Note**: ^§^, Wilcoxon signed-rank test is performed. *, *p* < 0.05, **, *p* < 0.01.

**Table 5:** Participants’ satisfaction with the program.

Questions	Total, Median [IQR]	KM University Students (n = 13)	KMDs (n = 6)	Nurses (n = 3)	Other (n = 1)
*“How helpful were this mindfulness program and the smartphone application in your participation in the program?”* (0–10-point Likert)	8 [8, 9]	8 [8, 9]	8 [7.75, 8.5]	9 [8, NA]	7 [NA]
*“If there were a program like this in the future, would you be willing to participate?”* (1–5-point Likert)	4 [4, 5]	4 [4, 5]	4.5 [3.75, 5]	4 [4, NA]	4 [NA]
*“Are you willing to continue using the provided smartphone application?”* (1–5-point Likert)	4 [4, 5]	4 [4, 5]	4.5 [4, 5]	4 [4, NA]	4 [NA]
*“If there were a program like this in the future, would you recommend it to your colleagues?”* (1–5-point Likert)	5 [4, 5]	5 [4, 5]	5 [4, 5]	4 [4, NA]	4 [NA]
*Are you willing to recommend the smartphone application provided to your colleagues?* (1–5 point Likert)	4 [4, 5]	4 [4, 5]	4 [4, 5]	4 [4, NA]	4 [NA]

**Abbreviations**: IQR, interquartile range; KM, Korean medicine; KMD, Korean medicine doctor; NA, not applicable.

## Data Availability

The data presented in this study are available on request from the corresponding author. The data are not publicly available due to privacy and ethical restrictions related to participant confidentiality.
